# Daily and Estral Regulation of RFRP-3 Neurons in the Female Mice

**DOI:** 10.5334/jcr.212

**Published:** 2021-04-15

**Authors:** Eleni Angelopoulou, Perrine Inquimbert, Paul Klosen, Greg Anderson, Andries Kalsbeek, Valérie Simonneaux

**Affiliations:** 1 University of Strasbourg, France; 2 University of Otago, New Zealand; 3 University of Amsterdam, Netherlands; 4 CNRS, France

**Keywords:** daily rhythm, female reproduction, arginin-vasopressin, RFRP-3, estrogen

## Abstract

Female reproductive success relies on proper integration of circadian- and ovarian- signals to the hypothalamic-pituitary-gonadal axis in order to synchronize the preovulatory LH surge at the end of the ovarian follicular stage with the onset of the main active period. In this study, we used a combination of neuroanatomical and electrophysiological approaches to assess whether the hypothalamic neurons expressing Arg-Phe amide-related peptide (RFRP-3), a gonadotropin inhibitory peptide, exhibit daily and estrous stage dependent variations in female mice. Furthermore, we investigated whether arginine vasopressin (AVP), a circadian peptide produced by the suprachiamatic nucleus regulates RFRP-3 neurons. The number of c-Fos–positive RFRP-3 immunoreactive neurons is significantly reduced at the day-to-night transition with no difference between diestrus and proestrus. Contrastingly, RFRP neuron firing rate is higher in proestrus as compared to diestrus, independently of the time of the day. AVP immunoreactive fibers contact RFRP neurons with the highest density observed during the late afternoon of diestrus and proestrus. Application of AVP increases RFRP neurons firing in the afternoon (ZT6-10) of diestrus, but not at the same time point of proestrus, indicating that AVP signaling on RFRP neurons may depend on circulating ovarian steroids. Together, these studies show that RFRP neurons integrate both daily and estrogenic signals, which downstream may help to properly time the preovulatory LH surge.

## Introduction

Mammalian reproductive competence is controlled centrally by neuroendocrine mechanisms operating along the hypothalamic- pituitary- gonadal (HPG) axis. In female mammals, the HPG axis is submitted to a complex regulation leading to a time-controlled ovulation triggered by an acute and transient luteinizing hormone (LH) release from the pituitary gland. This LH surge is driven by a former gonadotropin-releasing hormone (GnRH) surge which depends on high circulating estrogen (E2) secreted by mature ovarian follicles [1,2]. The preovulatory GnRH/LH surge also relies on a functional circadian system which coordinates the timing of ovulation and sexual behavior, ensuring that these events occur when the animal is most likely to breed [3,4,5]. Thus, in female nocturnal rodents, like mice, the LH surge is generated exclusively at the transition between the inactive (light) and the active (dark) periods at the proestrus stage when oocytes reach maturation [5,6,7]. E2 does not feedback directly on GnRH neurons which lack E2 receptors (ERa) [7,8] but rather on two hypothalamic ERa-expressing neuropeptidergic systems synthesizing the stimulatory kisspeptin (Kp) [9] and the inhibitory (Arg)(Phe) amide-related peptide-3 (RFRP-3) [10,11]. Kp and RFRP-3 neurons lie upstream of the GnRH neurons and are thought to be the converging site where daily and ovarian signals are conveyed to the HPG axis, therefore establishing the gated time window during which the preovulatory GnRH/LH surge occurs [12].

There are two hypothalamic Kp populations, one in the arcuate nucleus (ARC) and the other in the anteroventral periventricular nucleus (AVPV), which project to several hypothalamic regions and notably to GnRH neurons [13,14]. Many studies have shown that Kp administration stimulates GnRH neuron activity [15,16] and LH secretion in female mammals [17,18,19]. AVPV Kp neurons are considered pivotal for timing the preovulatory LH surge as they exhibit an increased neuronal activity and *Kiss1* expression at the day/night transition at the time of the LH surge [20,21,22,23]. RFRP-3 is the mammalian ortholog of the avian gonadotropin-inhibitory hormone (GnIH) [24,25]. RFRP-3 neurons are located in the dorsomedial hypothalamus (DMH) and project to multiple brain regions but most notably to GnRH neurons [10,26,27,28,29] and to Kp neurons [27,30] that express the RFRP-3 receptor, GPR147. Numerous studies have shown that RFRP-3 inhibits GnRH neuron activity and suppresses the elevated preovulatory LH release in female mammals [10,21,24,31,32,33,34,35,36]. Notably, in female rodents, RFRP-3 neuronal activity decreases at late day, at the time of the LH surge [21,22,37].

Studies have investigated the mechanisms through which circadian cues are relayed to AVPV Kp and RFRP-3 neurons, notably from the biological clock located in the suprachiasmatic nucleus (SCN) [20,21,37]. It is now well established that SCN-generated arginine vasopressin (AVP) fibers make appositions to AVPV Kp neurons which express V1a receptors [38,39], and that AVP activates Kp neurons in an E2-dependant manner [40]. Contrastingly, not much attention has been given to the circadian regulation of the RFRP-3 neurons. One study in Syrian hamsters showed that AVP- and vasoactive intestinal peptide (VIP)- ergic fibers originating from the SCN form close appositions to RFRP-3 neurons [41].

The aim of the present study was to investigate how the RFRP-3 system is regulated by daily and estrogenic cues in the female C57BL/6J mice. In a first set of neuroanatomical experiments, we analyzed whether there are daytime- and estrous cycle stage-dependent changes in the RFRP-3 expression and neuronal activity, and in the number of AVP-ergic fiber projections on RFRP-3 neurons. In a second set of electrophysiological experiments, we investigated the effect of daytime and estrous status on the RFRP-3 electrical activity and whether AVP regulates electrical activity of RFRP-3/EYFP fluorescent protein labelled neurons in hypothalamic sections of transgenic RFRP/Cre female mice.

## Materials and Methods

### 1. Animals

Adult female C57BL/6J mice (Charles River, France) and heterozygous RFRP-Cre mice on a C57BL/6J background [42] were housed two or three per cage under a 12-hour light: 12-hour dark cycle (lights on at 7:00 am, given as zeitgeber 0 (ZT0)) with controlled temperature (22°C) and *ad libitum* access to food and water. All protocols were reviewed by the Regional Committee for Ethics in Animal Experimentation and approved by the French Ministry of Education and Research (authorization #8452-2017010613574177v2).

### 2. Monitoring of Estrous Cycle and LH Secretion

Female mice were studied at either proestrus or diestrus stage as assessed by vaginal smears and circulating LH. Vaginal smears were performed daily at ZT2 during at least two consecutive cycles and only mice showing regular 4–5 day cycles were sampled on diestrus (appearance of leucocytes) or proestrus (appearance of nucleated epithelial cells). From each mouse a 4 µl blood sample was collected from the tail tip at the moment of sacrifice and this sample was diluted in 116 µL phosphate buffer saline with 0,25% tween-20. The LH concentration was measured by ELISA using anti-bovine LHβ as capture antibody (monoclonal antibody, 518B7, NHPP, Torrance, California), rabbit anti-mouse LH as first antibody (polyclonal antibody, rabbit LH antiserum, AFP240580Rb, NHPP), goat anti-rabbit IgG as secondary antibody (D048701–2, Dako Cytomation, Polyclonal Goat Anti-Rabbit, Denmark) and mouse LH as standard (mLH, AFP-5306A, NHPP) as previously described [43].

### 3. Neuroanatomical Investigation of Daily and Estral Regulation of RFRP-3 Neurons

#### Tissue processing

Adult female mice at proestrus or diestrus stage were sacrificed by exposure to increasing concentration of CO_2_ at six different time points (ZT0, ZT4, ZT8, ZT12, ZT16 and ZT20; n = 5 per experimental point). After intracardiac blood puncture, mice were intracardially perfused with 10 mL phosphate buffer saline 0.1 M (PBS, pH 7.4) followed by 20 mL of periodate-lysine-paraformaldehyde fixative (formaldehyde 4%, NaIO_4_ 10 mM and lysine 75 mM in 100 mM phosphate buffer, pH 7.3). The brains were collected, post-fixated in periodate-lysine-paraformaldehyde for 12 hours, washed out with PBS, dehydrated and embedded in polyethylene glycol as previously described [44].

Twelve series of 12 μm-thick coronal brain sections were cut using a microtome throughout the DMH as presented in the Paxinos mouse brain atlas. For each mouse, one section in every twelve (i.e. 1 section every 144 µm giving 6–7 DMH-containing brain sections) was rehydrated and mounted on a SuperFrost Plus (Menzel-Glaser, Braunschweig, Germany) slide. For each immunolabeling, DMH-containing slices of all mice of different time points and estrous stages were processed at the same time in order to limit variations in the labeling background.

#### Double c-Fos/RFRP-3 and AVP/RFRP-3 immunohistochemistry

The number of c-Fos expressing RFRP-3 neurons and the density of AVP fibers surrounding RFRP-3 neurons were assessed by dual immunohistochemistry. Brain sections were first incubated either with a rabbit polyclonal antiserum raised against c-Fos (1:2000; Santa Cruz Biotechnology; RRID: AB_627251) or a rabbit polyclonal antiserum raised against neurophysin II, a cleavage product of prepro-vasopressin (1:15000; Sigma-Aldrich; RRID: AB_260747) diluted in 154 mM PBS buffer containing 10% donkey serum and 0.3% Tween 20, for 24 hours at room temperature. Brain sections were washed with PBS, incubated with biotinylated donkey antirabbit (1:2000; Jackson Labs; in 154 mM PBS buffer containing 10% donkey serum and 0.3% Tween 20) for 1 hour and then washed again with PBS. Immunoreactive signal was amplified by a treatment with the avidin biotin complex coupled to peroxidase (1:250; Vector Laboratories) for 1 hour, then revealed using a solution of 0.5 mg/mL 3,3-diaminobenzidine (DAB; Sigma-Aldrich) diluted in water and 0.001% hydrogen peroxide urea (Sigma-Aldrich) for 30 minutes. Before performing the second immunolabelling, the antibodies were eluted with 2 x 15 minute washes in a solution of 100 mM glycine containing 0.3% Triton X-100 (pH 2.2). Brain sections were then incubated with a primary antibody directed against RFRP-3 (1:15000; rabbit anti-RFRP-3, University of Otago, NZ; RRID: AB_2877670; in a PBS buffer containing 10% donkey serum and 0.3% Tween 20) overnight at room temperature. The sections were then washed with PBS, incubated with biotinylated donkey anti-rabbit (1:2000; Jackson Labs; in 154 mM PBS buffer containing 10% donkey serum and 0.3% Tween 20) for 1 hour, and then washed again with PBS. The RFRP-3 signal was detected using streptavidin-peroxidase at 1/3000 (Roche) for 1 hour and revealed with Fast blue- BB (Sigma-Aldrich) for 7 minutes. Finally, sections were mounted with CC/mount (Sigma-Aldrich), dehydrated in toluene twice for 10 minutes, and mounted with Eukitt resin (Sigma-Aldrich).

#### Quantification of immunolabeled RFRP-3 cells and AVP-ergic fibers

Only five sections located at comparable levels of the DMH were taken into account for the analysis in order to limit differences stemming from rostrocaudal variations. The five sections were selected based on neuroanatomical markers such as the median eminence, the tuberoinfundibular sulcus and the pituitary stalk. Counting was done by the first author unaware of animal’s identity. For each mouse, the total number of RFRP-3-immunoreactive (ir) neurons, the number of RFRP-3 neurons containing nuclear c-Fos, and the number of RFRP-3 neurons receiving direct AVP-fiber projections were counted manually. AVP-ergic appositions were defined as terminal fibers directly contacting RFRP-3 cell somas. For each mouse, the number of RFRP-3 neurons is given as the total number counted in 5 sections. The number of c-Fos expressing RFRP-3 neurons is given as a percentage of this total number of RFRP-3 neurons. Also, the number of RFRP-3 neurons with close AVP-fiber appositions is given as a percentage of the total number of RFRP-3 neurons.

The density of AVP-ir fibers was quantified in selected regions of interest (ROI) bilaterally in the DMH defined by neuroanatomical landmarks (such as the median eminence, the tuberoinfundibular sulcus and the pituitary stalk), the DMH boundaries and the anatomical position of the RFRP-3 neurons. The density of AVP-fibers in the DMH was estimated by counting manually the number of points that a fiber crossed the intersections of a grid applied on the ROIs. For each mouse AVP-ir fiber density is given as total number of crossing points/total grid number of the ROIs.

### 4. Electrophysiological Investigation of Daily and Estral Regulation of RFRP-3 Neurons

#### In vivo labelling of RFRP-3 neurons

Labelling of RFRP neurons in adult female mice was performed using an adeno-associated virus containing a promoter upstream of a transcription-blocking cassette, followed by sequences encoding enhanced yellow fluorescent protein (pAAV5.EF1a.DIO.EYFP, 1 × 10^13^ pfu/mL, purchased from Addgene, catalog 27056-AAV5) in order to mediate the expression of EYFP exclusively in Cre-expressing RFRP neurons. Female RFRP-Cre mice were anaesthetized under a Zoletil (80 mg/kg)/Xylazine (10 mg/kg) mixture and injected with Metacam (5 mg/kg) and Bupivacaine (2 mg/kg) for analgesia. Then mice were placed in a stereotaxic apparatus in order to perform a bilateral stereotaxic injection of the virus targeting the DMH area. The skull was exposed and a Hamilton syringe loaded with 1 μL of AAV-EYFP was lowered into the DMH area according to the atlas of Paxinos and Franklin coordinates (–1.6 mm posterior to bregma, –0.5 mm lateral to midline, and –5.3 mm ventral to dura.). Injection was performed at a rate of 100 nL/min followed by a 10-minute pause before removing the syringe. Mice were administered Metacam (2 mg/kg) dissolved in water for 3 days as a post-operative analgesic treatment. Preliminary tests indicated that the highest level of EYFP expression in RFRP-3 neurons was observed three weeks after the injections with the percentage of RFRP-3-ir neurons expressing EYFP estimated at 68.3 ± 2.5 % (n = 4 mice).

#### Brain slice preparation

Three weeks after the AAV injections, mice were anaesthetized with urethane (i.p. 1.9 g/Kg) at two estrous stages (diestrus or proestrus) and two different time points ZT4-ZT5 (*in vitro* recording at ZT6-ZT10) or ZT9-ZT10 (*in vitro* recording at ZT11-ZT14, time of the preovulatory LH surge at the proestrus stage). An intracardic perfusion was performed with oxygenated iced-cold sucrose-artificial cerebrospinal fluid (sucrose-aCSF, containing 248 mM sucrose, 11 mM glucose, 26 mM NaHCO3, 2 mM KCl, 1.25 mM KH2PO4, 2 mM CaCl2, 1.3 mM MgSO4, 5 mM kynurenic acid). The solution was continuously bubbled with 5% CO2 and 95% O2. Brains were quickly removed and 300 μm thick coronal slices were cut at the level of the DMH using a vibratome (Leica VT1200S). Slices were then transferred in artificial cerebrospinal fluid (aCSF) containing 126 mM NaCl, 26 mM NaHCO3, 2.5 mM KCl, 1.25 mM NaH2PO4, 2 mM CaCl2, 2 mM MgCl2, 10 mM glucose at room temperature for 1 hour before starting recordings.

#### RFRP cell-attached recordings

After recovery, slices were placed to the recording chamber under an upright microscope fitted for epifluorescence (Zeiss, Axioskop) and were continuously perfused with oxygenated aCSF (1.0–1.5 mL/min). EYFP/RFRP neurons were first visualized by brief fluorescent illumination. Spontaneous firing was recorded using the minimally invasive cell-attached loose patch configuration. Recording electrodes (3–5 MΩ) were pulled from borosilicate glass capillaries (Harvard apparatus) using a P1000 electrode puller (Sutter Instruments) and filled with aCSF. Spontaneous spikes were recorded in the voltage clamp mode using an Axopatch 200B amplifier (Axon Intruments). Signals were low-pass filtered (5 kHz) and acquired with Clampex software (Molecular devices). Current traces were digitized (10 kHz) and stored on the hard drive of a personal computer. All experiments were performed at room temperature (22–24 °C). The spontaneous firing of neurons was recorded in control aCSF for more than 5 minutes before bath application of AVP (1 µM in aCSF; Euromedex) for 3 minutes. Spontaneous spikes were detected using the threshold-crossing method in pClamp 10 (Molecular device). Data calculated were the Mean Interspike Interval (ISI) and the Coefficient of Variation of ISI (CV = standard deviation/mean ISI) as an indicator of spike train regularity. Recordings in which the baseline firing was not stable were not included in the analysis.

### 5. Data Analysis

Statistical analysis was performed with GraphPad Prism 6. All data, including outliers, have been included in the analyses. All values are given as mean ± SEM. For neuroanatomical results, Two-way ANOVA was used to assess significant variations among different time points and estrous stages, followed by Sidak’s multiple comparisons test. For the electrophysiological investigation, the firing pattern of individual RFRP neurons was determined with criteria previously used for Kp neurons: silent neurons displaying no spontaneous activity, tonic neurons exhibiting regular firing with the SD of their interspike intervals less than 100 msec, bursting neurons having more than 50% of their spikes occurring in bursts, and all other neurons classified as irregular [45,46]. Two-way ANOVA was used to assess significant variations in the firing properties among different time points and estrous stages. For AVP experiments, the relative ISI (to a 5-minute baseline) was averaged in 1 minute-bin and drug effect was tested with repeated measures Two-way ANOVA (RM-ANOVA). Comparison of mean firing frequency between baseline and following AVP application was performed using a paired Student t-test. Differences were considered significant for P < 0.05.

## Results

### 1. Neuroanatomical Investigation of Daily and Estral Regulation of RFRP-3 Neurons in Female C57BL/6J Mice

**Figure 1:**
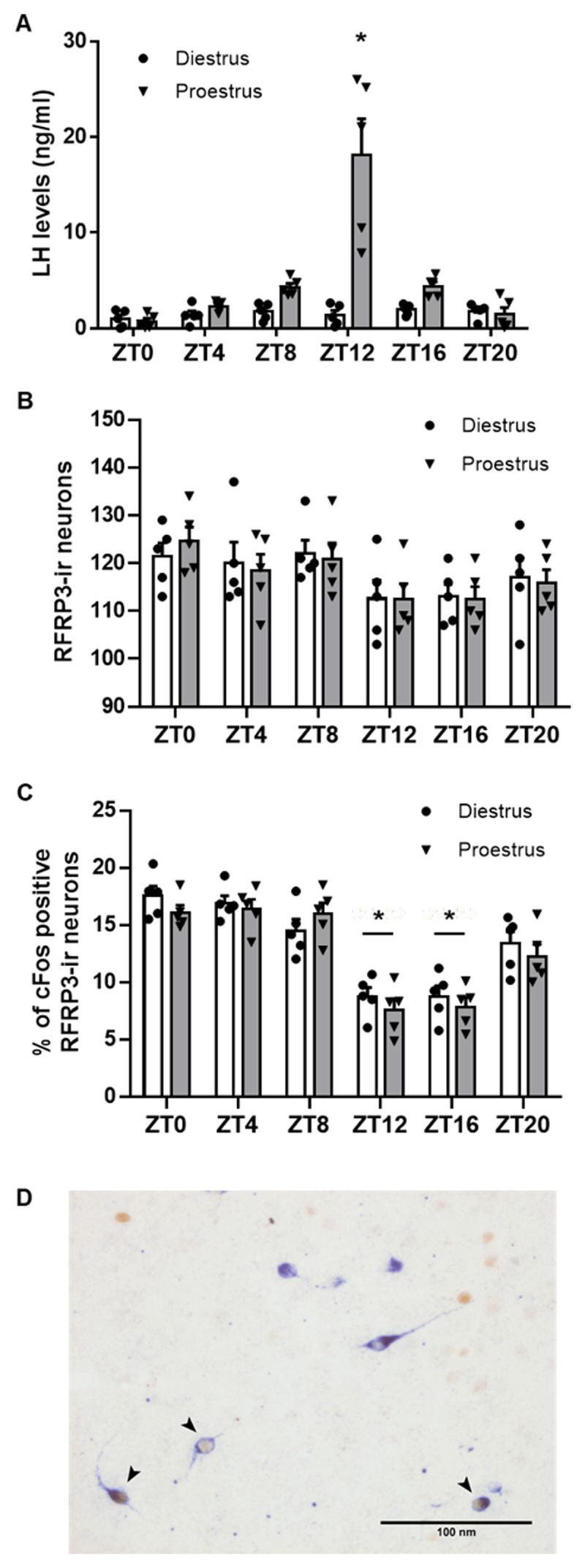
**RFRP-3 immunoreactivity and neuronal activity in the dorsomedial hypothalamus of female C57BL/6J mice sampled at different time points of the day of diestrus and proestrus. (A)** LH blood concentrations; **(B)** number of RFRP-3-ir neurons in the DMH; **(C)** percentage of c-Fos–expressing RFRP-3-ir neurons; **(D)** Photograph where arrows show c-Fos–positive (brown) RFRP3-ir (blue) neurons at ZT0 (08:00 am) on the day of proestrus; scale bar = 100 µm. Data are presented as mean ± SEM of n = 5 mice for each experimental point. Two-way ANOVA was used to assess significant variations among different time points and estrous stages, followed by Sidak’s multiple comparisons test. Daily differences with a value of P < 0.05 were considered as significant and are indicated by *. Cycle stage differences were insignificant (P > 0.05).

This experiment aimed at investigating the occurrence of daily and estral variation in RFRP-3 expression, neuronal activity and inputs from AVP-ergic fibers in female mice. Mice sampled at different time points of the proestrus stage exhibited the expected surge in LH at ZT12, whereas mice sampled at the diestrus stage had constant low LH levels at all timepoints (Two-way ANOVA, estrous stage, F (1, 48) = 29.97, p < 0.0001; daytime, F (5, 48) = 15.60, p < 0.0001; ***Figure 1A***).

Quantification of the number of RFRP-3 neurons in the DMH showed no changes in the number of RFRP3-ir neurons at the different time points of proestrus and diestrus (Two-way ANOVA, estrous stage, F (1, 48) = 0.01923, p = 0.8903; daytime, F (5, 48) = 3.518, p = 0.0086; ***Figure 1B***). By contrast, the percentage of c-Fos-positive RFRP-3 neurons was significantly reduced at the day-to-night transition (ZT12-ZT16), thus around the time of the LH surge, on the day of proestrus, but also at the same period on the day of diestrus (Two-way ANOVA, daytime, F (5, 48) = 39.56, p < 0.0001; estrous stage, F (1, 48) = 1.413, p = 0.2403; ***Figure 1C, Figure 1D***).

**Figure 2:**
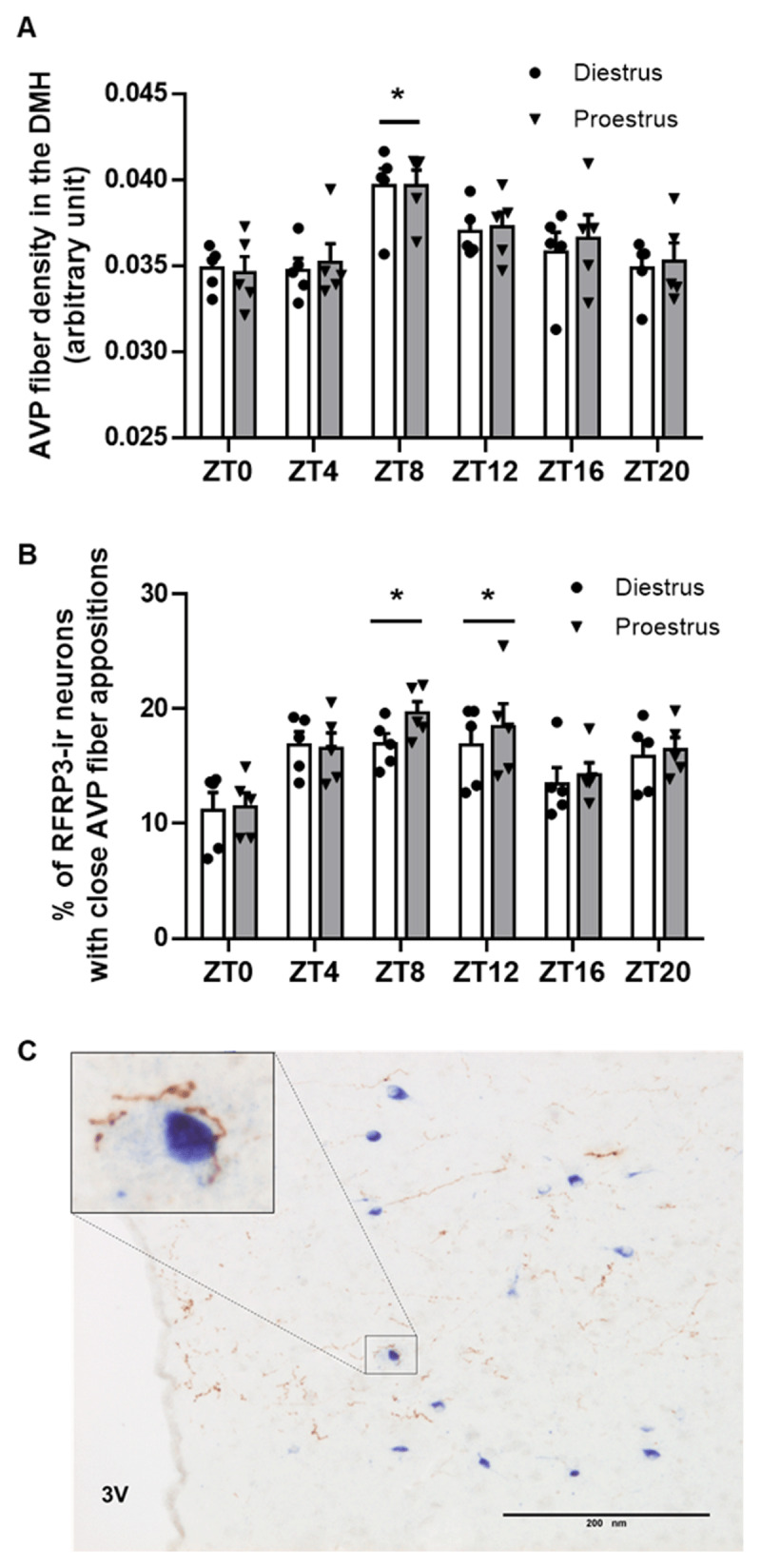
**Distribution of arginine vasopressin (AVP)-immunoreactive (ir) fibers in the dorsomedial hypothalamus of female C57BL/6J mice sampled at different time points of the day of diestrus and proestrus. (A)** AVP-ir fiber density (arbitrary unit) in the DMH; **(B)** Percentage of RFRP-3-ir neurons with close AVP-ir fiber appositions; **(C)** Photograph shows RFRP-3-ir neurons and AVP-containing fibers at ZT8 (16:00) on the day of proestrus; scale bar = 200 µm. Data are presented as mean ± SEM of n = 5 mice for each experimental point. Two-way ANOVA was used to assess significant variations among different time points and estrous stages, followed by Sidak’s multiple comparisons test. Daily differences with a value of P < 0.05 were considered as significant and are indicated by *. Cycle stage differences were insignificant (P > 0.05).

Analysis of AVP-ir fiber density in the DMH showed a significant increase at ZT8, thus prior to the onset of the dark period, with no difference between proestrus and diestrus (Two-way ANOVA, daytime, F (5, 48) = 7.759, p < 0.0001; estrous stage, F (1, 48) = 0.2844, p = 0.5963; ***Figure 2A***). Quantification of the percentage of RFRP-3 neurons receiving direct AVP-ir fiber appositions showed that the highest percentage is observed at ZT8-ZT12 and the lowest percentage at ZT0, with no difference between proestrus and diestrus (Two-way ANOVA, daytime, F (5, 48) = 7.673, p < 0.0001; estrous stage, F (1, 48) = 1.467, p = 0.2318; ***Figure 2B, Figure 2C***).

### 2. Electrophysiological Investigation of Daily and Estral Regulation of RFRP-3 Neuron Firing Activity in Female C57BL/6J Mice

**Figure 3:**
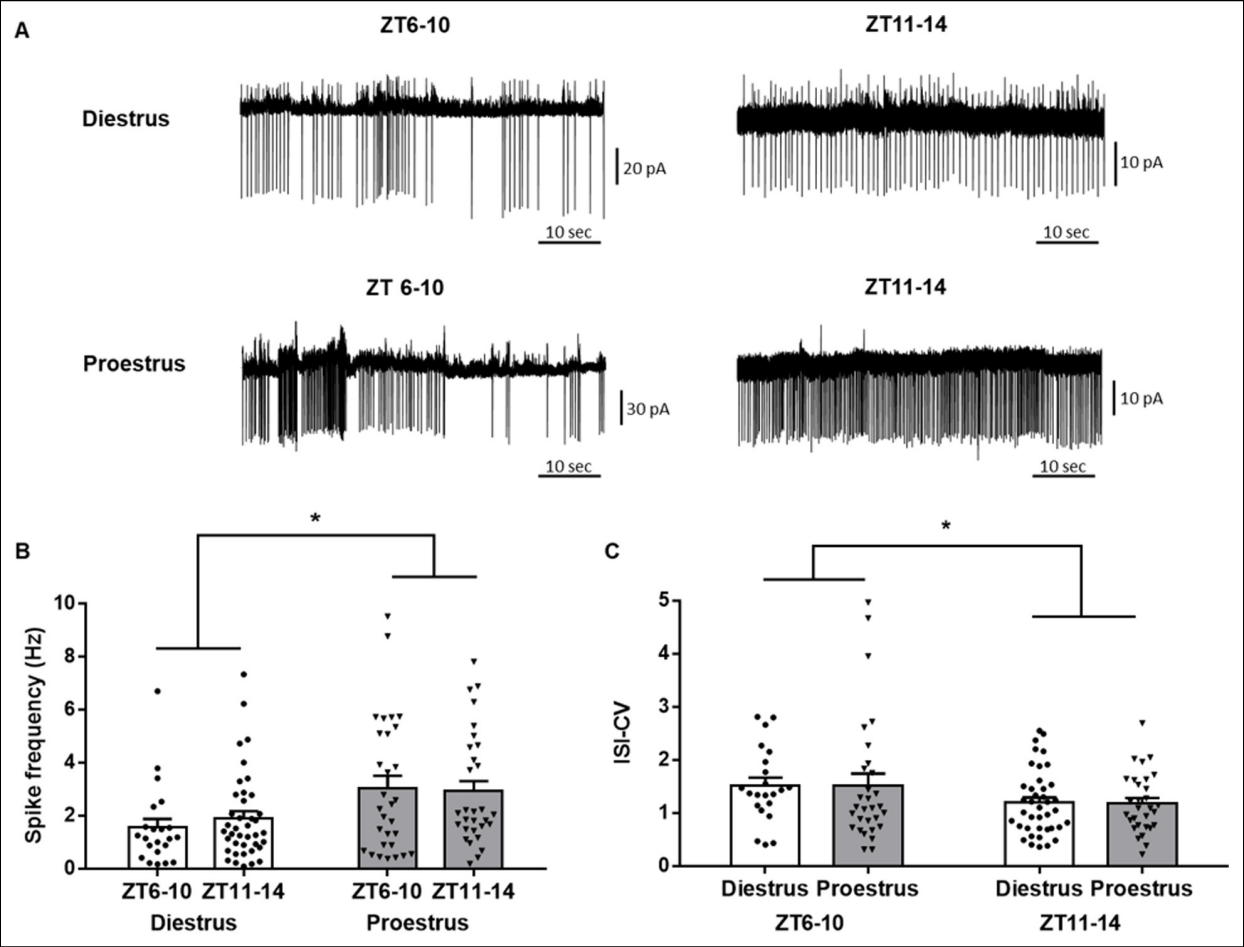
**Electrical firing characteristics of RFRP-3 neurons in the dorsomedial hypothalamus at two time points and stages of the estrous cycle in female C57BL/6J mice. (A)** Representative traces of RFRP-3 neurons firing in the DMH at two time points (ZT6-10 and ZT11-14) during diestrus and proestrus; **(B)** Mean spike frequency of RFRP-3 neurons in the DMH at two time points during the day of diestrus (ZT6-10; n=22 neurons out of 7 mice and ZT11-14; n=42 neurons out of 10 mice) and proestrus (ZT6-10; n=30 neurons out of 7 mice and ZT11-14; n=30 neurons out of 9 mice); **(C)** InterSpike Interval CV (ISI-CV) of RFRP-3 neurons in the DMH at two time points during the day of diestrus and proestrus. Data are presented as mean ± SEM. Two-way ANOVA was used to assess significant variations in the firing properties among different time points and estrous stages. Differences among groups were considered significant for P < 0.05 and are indicated by *.

This experiment aimed at recording the endogenous firing activity of RFRP-3 neurons at two time points of the diestrus and proestrus stages, and its regulation by AVP at these different experimental points. All recorded RFRP-3 neurons exhibited an irregular spontaneous firing pattern (***Figure 3A***). RFRP-3 neuron spontaneous firing rates were significantly higher in proestrus compared to diestrus (Two Way ANOVA, estrous stage, F (1, 118) = 11.45, p = 0.0010) independently of the time of the day (Two Way ANOVA, daytime, F (1, 118) = 0.1032, p = 0.7486; ***Figure 3A, Figure 3B***). Similarly, on the day of proestrus, we recorded a lower InterSpikeInterval, indicating a shorter time between subsequent action potentials in RFRP-3 neurons (Two Way ANOVA, estrous stage, F (1, 118) = 6.288, p = 0.0135), independently of the time of the day (Two Way ANOVA, daytime, F (1, 118) = 2.067, p = 0.1532, data not shown).

In contrast, the InterSpikeInterval-Coefficient of Variation (ISI-CV), indicator of firing regularity, was lower at ZT11-14 compared to ZT6-10 (Two Way ANOVA, daytime, F (1, 118) = 5.021, p = 0.0269; ***Fig. 3c***) independently of the estrous stage (Two Way ANOVA, estrous stage, F (1, 118) = 0.00077, p = 0.9780; ***Figure 3C***), thus indicating that RFRP-3 neurons have a more regular firing pattern at ZT11-14 compared to the earlier period.

**Figure 4:**
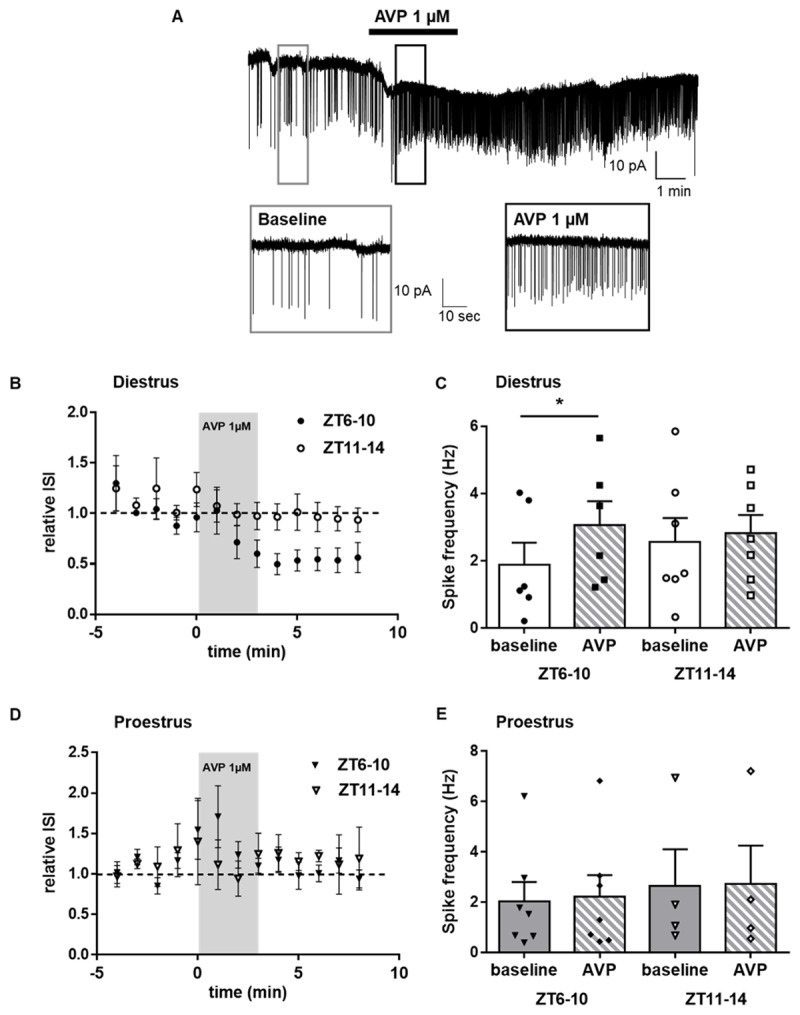
**Effect of AVP on RFRP-3 electrical activity in the dorsomedial hypothalamus at different time points and stages of the estrous cycle in female C57BL/6J mice. (A)** Sample trace illustrating the effect of AVP on RFRP-3 neuron firing in Diestrus at ZT6-10. **(B)** Average time course of relative InterSpikeInterval (ISI) in response to AVP (1µM) application of RFRP-3 neurons in the DMH at ZT 6-10 (n=6 neurons out of 5 mice) and ZT11-14 (n=7 neurons out of 3 mice) during the day of diestrus. **(C)** Summary bar graph of the AVP effect on mean spike frequency of RFRP-3 neurons in the DMH during the day of diestrus. **(D)** Average time course of relative InterSpikeInterval (ISI) in response to AVP (1µM) application of RFRP-3 neurons in the DMH at ZT 6-10 (n=7 neurons out of 5 mice) and ZT11-14 (n=4 neurons out of 3 mice) in the day of proestrus. **(E)** Summary bar graph of the AVP effect on mean spike frequency of RFRP-3 neurons in the DMH the day of proestrus. Data are presented as mean ± SEM. Repeated measures Two-way ANOVA was used to assess significant variations in the relative ISI among different time points in diestrus and proestrus. A paired Student t-test was used to compare RFRP mean firing frequency between baseline and after AVP application. Differences among groups were considered significant for P < 0.05 and are indicated by *.

In diestrus mice, bath application of AVP (1µM) at ZT 6-10 significantly decreased the ISI (RM-ANOVA for relative ISI, time, F (12, 132) = 3,092, p = 0.007, n = 6; ***Figure 4A, Figure 4B***) and consequently increased the mean firing rate of RFRP-3 neurons (paired Student’s t-test, p = 0.0199, n = 6; ***Figure 4C***), but had no effect at ZT11-ZT14. In proestrus mice, AVP (1µM) application had no effect on the ISI (RM-ANOVA, for relative ISI, time, F (12, 108) = 1.023, p = 0.4330, n = 7; ***Figure 4D***) and so neither on the mean firing rate of RFRP-3 neurons (paired Student’s t-test, time, p = 0.2840, n = 7; ***Figure 4E***) at either time point.

## Discussion

Reproduction in female mammals displays regular daily and ovarian cycles driven by E2-sensitive neural circuits in the hypothalamus [47,48,49]. In this study, we investigated in female mice whether RFRP-3 neurons, reported to regulate GnRH neuronal activity and LH secretion [12], act as an interface for SCN-derived AVP fibers and E2 feedback, in order to convey daily and estral cues to the HPG axis. Therefore, we examined whether the RFRP-3 system is under daily regulation and/or sensitive to estrogenic cues, as previously reported for Kp neurons [48,50]. Using a neuroanatomical approach, we report a decrease in RFRP-3 neuron activation coordinated with an increase in the number of RFRP3-ir cells with close AVP-ir fiber appositions around the day-to-night transition (ZT8-ZT16) with no difference between the diestrus and proestrus stages. Using electrophysiological analysis, we report that RFRP-3 neurons display an increased firing rate in proestrus as compared to diestrus and a more regular firing activity during the day-to-night transition (ZT11-14) as compared to midday (ZT6-10). Moreover, AVP affects the activity of RFRP-3 neurons in a time- and estrous stage-dependent way, with a stimulatory effect observed at midday of the diestrus day only. It is worth saying that in a preliminary experiment, we found a low density of VIP- containing fibers (another SCN-derived input on RFRP-3 neurons; [41,51] in the DMH, and the VIP-ergic fiber input on RFRP-3 neurons showed no daily or estral variation, while VIP was unable to alter the firing rate of RFRP-3 neurons (unpublished data).

In this study, we assessed RFRP-3-immunoreactivity and neuronal activity, both by c-Fos expression and by electrophysiological recordings, at different time points of diestrus and proestrus. We found no daily- or estrous stage- dependent changes in RFRP3-ir cell numbers, but, we observed that RFRP-3 neuronal activation, as attested by c-Fos expression, decreases during the day-to-night transition of both proestrus and diestrus day, as previously reported in female Syrian hamsters [21,37]. Therefore, on proestrus, there is a coordinated decrease in RFRP-3 neuronal activity with a concurrent increase in Kp neuronal activity and LH surge [20,21,23] at the day-to-night transition, whereas on diestrus, only RFRP-3 neurons exhibit a daily variation. Thus, the daily pattern in RFRP-3 c-Fos activation appears independent to the stage of the estrous cycle, even though it has been reported that a subset of RFRP-3 neurons express ERα, and that E2 administration suppresses RFRP expression [11,52,53]. When investigating the electrical activity of RFRP-3 neurons in isolated hypothalamic sections, we observed that they exhibit different firing activities according to the time of the day and the estrous stage. Indeed, we calculated that the RFRP-3 neuron firing activity is more regular during the day-to-night transition compared to an earlier time period, in both estrous stages. However, RFRP-3 neurons fire action potentials at an about twice higher frequency in proestrus compared to diestrus with no time of the day difference.

Altogether, both neuroanatomical and electrophysiological results indicate that RFRP-3 neurons may be involved in the daily and estral regulation of the HPG axis. Yet, data acquired by means of immunohistochemistry and electrophysiology display some differences. Notably, we report a clear estrous stage difference in the intrinsic firing activity but not in c-Fos expression of RFRP-3 neurons. The inducible immediate early gene *c-fos* is a reporter of transcriptional activation widely used as a molecular marker of neural activity, although the threshold of c-Fos induction differs among different neural populations. In some brain areas c-Fos is expressed after exposure to mild stimulus, in other brain structures a previous stimulus deprivation is necessary, and certain active neural populations cannot elicit sufficient c-Fos expression [54,55]. Furthermore, c-Fos expression is triggered by different signals, among which an increase in calcium influx, but interestingly, calcium influx during spike activity cannot induce c-Fos expression [56]. Therefore, given our observation that RFRP-3 neurons are constantly spontaneously active, it is possible that the higher frequency observed at proestrus does not translate in a different c-Fos expression profile. However, it is important to note that c-Fos expression is not a reliable marker of neuronal activity, since its induction is not associated with firing rate but rather with the neuronal firing pattern [57].

Female reproduction relies on a synchronized circadian system to properly time the preovulatory LH surge as SCN lesions and genetic impairment of clock genes suppress the LH surge and lead to estrous acyclicity [4,58,59]. A significant amount of evidence links the circadian peptide AVP with the SCN-driven daily regulation of the HPG axis. AVP central injections restore the LH surge in rodents with SCN lesions or clock gene mutation, while central administration of AVP receptor antagonists suppresses the LH surge [60,61,62]. Neuroanatomical, pharmacological and electrophysiological data have proven that the SCN-derived AVP input on AVPV Kp neurons is critical for the LH surge induction [38,39,40]. However, an earlier study in Syrian hamsters showed that SCN-derived AVP neurons also make close appositions with RFRP-3 neurons, although central administration of AVP had no effect on the RFRP-3 c-Fos expression [41]. We confirmed, in female mice, that RFRP-3 neurons receive close AVP-ergic fiber appositions, and we furthermore reported a significant increase in the number of RFRP-3 neurons with close AVP-ergic fiber appositions at late afternoon, concurrently with an increase in the AVP-ergic fiber density in the DMH. Thus, prior to the light-dark transition, and in line with higher levels of AVP mRNA in the SCN [51], there is an increased input of AVP signaling on RFRP-3 neurons. Notably, the overall AVP-ergic fiber input to RFRP-3 neurons is similar in both diestrus and proestrus, which might reflect the E2 independent daily rhythms in SCN AVP synthesis [63]. The presence of peptidergic fiber appositions on neuronal cell bodies does not prove that they make synapses or that there is a direct post-synaptic effect of the peptide. Therefore, in order to look for a functional role of AVP on RFRP-3 neurons, we examined its effect on RFRP-3 neuronal firing activity either at ZT6-10 or at ZT11-14 on diestrus and proestrus. We showed that AVP increases the RFRP-3 neuron firing frequency at midday and not at the day-to-night transition of diestrus, while it has no effect at both time points in proestrus. Our findings indicate that AVP may coordinate the RFRP-3 system in a specific time window under low E2 milieu (diestrus) and that E2 (at proestrus) may act in a suppressive manner by disabling the excitatory effect of AVP on RFRP-3 neurons. It is interesting to note that an earlier study also reported a role of E2 in the AVP-induced activation of AVPV Kp neurons [40]. Future experiments should evaluate whether RFRP-3 neurons express V1aR receptors and whether there are circadian- and E2-dependent changes in the V1aR co-expression that could mediate the above mentioned changes. Comparing our results in mice, which report a stimulatory effect of AVP, but not VIP, on RFRP-3 neurons firing rate, to the earlier study in Syrian hamsters, which showed that VIP suppressed the number of c-Fos expressing RFRP-3 neurons in the evening while AVP had no effect on the RFRP-3 c-Fos expression, [41], it might be worth investigating whether there are species-dependent differences in the effect of circadian peptides on RFRP-3 neurons.

## Conclusion

In conclusion, the current study adds to the evidence that RFRP-3 neurons are a converging site where daily and estrogenic signals are integrated and may be conveyed to the reproductive axis. Our current hypothesis is that the increased AVP input at late afternoon can induce an excitatory effect on the slow firing RFRP-3 neurons at diestrus, but is inefficient in further exciting the highly firing RFRP-3 neurons in proestrus. Given the inhibitory effect of RFRP-3 on LH secretion [24,35,52], our findings support that RFRP-3 neurons facilitate the gating of the LH surge at the right time of the day and stage of the estrous cycle. However, given that RFRP-3 is now reported to also regulate food intake [64] and stress response [42], we cannot exclude the possibility that RFRP-3 neurons may regulate female reproduction indirectly through metabolic- and stress-regulated mechanisms.
